# Assessing the potential impact of COVID-19 booster doses and oral antivirals: A mathematical modelling study of selected middle-income countries in the Indo-Pacific

**DOI:** 10.1016/j.jvacx.2023.100386

**Published:** 2023-09-09

**Authors:** Gizem M. Bilgin, Kamalini Lokuge, Syarifah Liza Munira, Kathryn Glass

**Affiliations:** a National Centre for Epidemiology and Population Health, The Australian National University, Acton, ACT 2601, Australia; b Faculty of Economics and Business, Universitas Indonesia, Jakarta, Indonesia

**Keywords:** COVID-19, SARS-CoV-2, Oral antiviral, Booster vaccine, Mathematical modelling, Middle-income

## Abstract

Continued efforts to reduce the burden of COVID-19 require the consideration of additional booster doses and emerging oral antivirals. This study explored the individual- and population-level impacts of booster dose and oral antivirals in Indonesia, Fiji, Papua New Guinea, and Timor-Leste. Our mathematical model included age structure, vaccine coverage, prevalence of comorbidities, and immunity from prior infection fit to incidence data from our study settings. We explored a range of eligibility criteria and found that boosters had the largest impact per dose when prioritised to high-risk adults and adults who had not previously received a booster. Antivirals were most effective in settings with low vaccine-derived immunity. In general, fewer antivirals than booster doses were required to prevent a hospitalisation or death. Only in settings with very high vaccine uptake was the impact per dose of providing booster doses to high-risk adults comparable to providing oral antivirals to high-risk adults. Together, booster doses and oral antivirals could prevent 80%, 64%, 49%, and 65% of deaths, and 38%, 37%, 16%, and 34% of hospitalisations in Fiji, Indonesia, Papua New Guinea, and Timor-Leste respectively. Therefore, our findings support the continued provision of COVID-19 booster doses to high-risk adults in 2023, and advocate for increased access to oral antivirals, especially in settings with low vaccine coverage such as Papua New Guinea. Future work should consider the threshold at which self-financing of COVID-19 oral antivirals would be viable for middle-income countries in South-East Asia and the Pacific.

## Highlights

• We modelled COVID-19 booster doses and antivirals in four middle-income countries.
• Our model included age, vaccination, comorbidities and immunity from prior infection.
• Providing COVID-19 booster doses to high-risk adults remains a priority.
• Oral antivirals will have a meaningful impact in all settings.

## Introduction

Increases in COVID-19 vaccine supplies have allowed most adults willing to be vaccinated in middle-income settings to complete their primary schedule. As of the 1st of January 2023, 139 and 213 doses per 100 individuals had been delivered in lower- and upper-middle income settings respectively [1]. Two options have emerged for mitigating severe outcomes associated with COVID-19 as we transition to endemicity: yearly booster doses, and antiviral treatments.

Oral antivirals present an emerging opportunity for reducing severe COVID-19 outcomes in low-resource settings. The World Health Organization (WHO) included a strong recommendation for the provision of oral antivirals to high-risk adults for the first time in April 2022, and expanded this strong recommendation to additional groups in January 2023 [2], [3]. Previously available antivirals, such as remdesivir and sotrovimab, required intravenous delivery, however newer oral antivirals provide the potential for early treatment and severe disease prevention in low-resource settings when supported by rapid antigen tests.

In this study, we use mathematical modelling to estimate the impact of oral COVID-19 antivirals and yearly COVID-19 booster doses on the burden of severe COVID-19 disease in Fiji, Indonesia, Timor-Leste, and Papua New Guinea during 2023. We have chosen these middle-income settings as case studies with varying vaccine coverage, vaccine hesitancy, prevalence of comorbidities, and age structure. We aim to understand how these setting characteristics influence the dose- and population-level impact of booster doses or antiviral programs in 2023. Our results are intended to inform strategies for distributing and advocating for COVID-19 booster doses or oral antivirals (supported by rapid antigen tests) to achieve a meaningful reduction of COVID-19 associated deaths and hospitalisations in middle-income settings. Considering the individual- and population-level impact of different eligibility strategies will be essential as we transition from pandemic to endemic approaches to managing COVID-19 and integrate COVID-19 management strategies with regular healthcare priorities.

## Methods

### Overview

We adapted a transmission model from our previous paper modelling COVID-19 vaccine prioritisation strategies in Sierra Leone (in preprint [4]) to four new settings: Fiji, Indonesia, Timor-Leste, and Papua New Guinea (Table 1). For this paper, we expanded the deterministic transmission model to include a stochastic component to model the effect of oral antivirals, with eligibility based on age and comorbidities.

**Table 1:** Comparison of key characteristics between study settings, visualised in Supplementary Material S1.6.

	**World Bank classification**[5]	**GDP per capita (USD 2021)**[5]	**Population**[6]	**Fertility rate in women 15 to 49 (%)**[6]	**Population over 60 (%)**[6]	**Population at high risk of severe COVID-19 (%)**[7]	**Primary schedule coverage (%) at 01/01/2023**[8]	**Primary schedule coverage in adults aged over 60 (%) at 01/01/2023**[8]
Fiji	Upper middle income	5,086	929,769	7.4	9.7	7.1	68.9	96.8
Indonesia	Upper middle income	4,292	275,501,336	6.3	10.9	4.7	62.6	50.4
Papua New Guinea	Lower middle income	2,916	10,142,625	9.7	5.5	4.7	3.1	2.5
Timor-Leste	Lower middle income	1,458	1,341,298	9.6	7.3	3.3	58.9	71.9

### Transmission model

In short, COVID-19 transmission was modelled using a deterministic Susceptible-Exposed-Infected-Recovered (SEIR) model stratified by age (0–4, 5–9,10–17, 18–29, 30–44, 45–59, 60–69, 70 + years), vaccination status, and prevalence of comorbidities. We reconstructed the vaccination history of each setting by dose and type, and estimated vaccine effectiveness by considering both homogenous and heterogenous combinations of primary and booster doses (Supplementary Material S1.1–2). Details of the fit of the transmission model to daily reported cases to estimate infection-derived immunity are provided in the Supplementary Material S1.3–5. We identified the age-specific distribution of individuals with comorbidities using age- and nation-specific estimates by Clark et al. of populations at high risk of severe outcomes (visualised Supplementary Material S1.6) [7]. We assumed that adults with comorbidities had 1.95 times (95% CI 0.99–3.82) higher risk of developing severe outcomes compared to adults without comorbidities [9].

### Target population for antivirals

We assumed that antiviral treatments would be given to symptomatic adults at high risk of severe disease, as per current WHO Guidelines for delivery of COVID-19 antivirals [3]. The WHO recommends the use of antivirals for individuals who are at the highest risk of hospitalisation including those of older age, who are immunosuppressed and/or have a chronic illness such as diabetes [3]. In line with this recommendation, we identified ‘high-risk adults’ as all individuals aged over 60 or aged 18–59 with a comorbidity associated with an increased risk of severe outcomes from COVID-19.

Our results include additional analysis for the delivery of antivirals to unvaccinated adults, pregnant women, and for the broader provision of antivirals to all symptomatic adults. The WHO guidelines list the lack of COVID-19 vaccination as an additional risk factor to consider when dispensing oral antivirals [3]. In January 2023, the WHO updated their therapeutic guidelines to permit the provision of COVID-19 antivirals to pregnant women [3]. Previously the WHO had not recommended the prescription of oral antivirals to pregnant women due to a lack of clinical data surrounding their safety [10]. Pregnant women had been excluded from large trials of oral antivirals including MOVe-OUT for molnupiravir (‘Lagevrio’), and EPIC-SR and HR for nirmatrelvir-ritonavir (‘Paxlovid’) [11], [12]. There is strong support for the consideration of use of antivirals in low- and middle-income countries where there are no other treatment options for pregnant women at high risk of severe disease [13].

### Antiviral effectiveness

We modelled the direct benefits of antivirals in reducing hospitalisation and death. We used real-world estimates for the effectiveness of oral antivirals from retrospective cohort studies (Table 2). These estimates were lower than previous estimates from stage 2/3 clinical trials, possibly due to longer delays from symptom onset to testing and subsequent access of antivirals, lower adherence to antiviral timing, and lower completion of the antiviral schedule in real-world studies compared to clinical trials.

**Table 2:** Antiviral parameters with point estimates, distributions, and sources.

**Parameter**	**Point estimate**	**Distribution**	**Source**
Nirmatrelvir-ritonavir effectiveness against death	88%	Beta (9.7,1.3)	[20]
Nirmatrelvir-ritonavir effectiveness against hospitalisation	68%	Beta (3.8,1.8)	[20]
Nirmatrelvir-ritonavir effectiveness against severe disease	57%	Beta (15.6,11.8)	[21]
Molnupiravir effectiveness against death	24%	Beta (6.2,19.7)	[22]
Molnupiravir effectiveness against hospitalisation	Not statistically significant	NA	[22]
Molnupiravir effectiveness against severe disease	30%	Beta (4.1,9.5)	[23]
Likelihood of testing positive within the treatment window	53.7%	27.1%-72.6%	[19]

We present our main results in this paper using effectiveness estimates for nirmatrelvir-ritonavir. We provide additional results for the use of molnupiravir in the Supplementary Materials as per the WHO’s recommendation on the use of the best oral antiviral dependent on availability [3]. Nirmatrelvir-ritonavir has numerous contraindications and interactions with drugs for the management of chronic conditions [14]. However, many of these chronic disease management medications for which nirmatrelvir-ritonavir is contraindicated (e.g. statins) can be temporarily paused, decreased or substituted to allow antiviral use without consequences for long-term disease management [15]. Additionally, and unfortunately, the reality is also that long-term access to essential medicines for the management of chronic conditions is limited in low- and middle-income countries due to low availability and unaffordability [16], [17]. A systematic review of the Asia Pacific found that the median availability of any medicine for chronic disease management was 35.5% in the public sector and 56.7% in the private sector [18].

The WHO recommendation for the use of oral antivirals acknowledges that rapid antigen testing (RAT) will need to be expanded to support oral antiviral deployment in low- and middle-income countries [3]. We assumed that all symptomatic adults have access to testing to estimate the possible population-level impact of oral antivirals. We used Menkir et al.’s estimate of the likelihood of testing positive to a RAT test within the treatment window of 5 days after symptom onset [19]. We assumed uniform effectiveness within this 5-day window based on nirmatrelvir-ritonavir clinical trial data assessing 88.9% relative risk reduction of patients commencing treatment within 3 days, compared to 87.8% within 5 days [12]. We assumed all individuals who received antivirals completed the full course of antivirals.

### Stochastic impact of antivirals

We separated the incidence of symptomatic disease from the transmission model into ‘individuals’ with distinct ages, vaccination status, prior immunity from infection, and dates of symptom onset. Then, we sampled the likelihood of these individuals testing positive within the treatment window. We estimated the outcomes averted by antivirals by considering the age and immunity profile of the cohort receiving antivirals. The likelihood of this cohort developing severe outcomes with and without antivirals was determined by sampling from distributions fitted to the 95% confidence intervals of infection-derived immunity, vaccine effectiveness, and antiviral effectiveness in each simulation, see available code for more detail.

### Configuration of simulations

We assumed that only individuals who had previously completed their primary schedule would be willing to be vaccinated with a booster dose. We also modelled booster ‘catch-up’ campaigns which targeted adults who had previously received a primary schedule but not a booster dose. To allow comparison between settings, we modelled booster programs as starting on the 1st of March 2023 with a constant speed of rollout – delivering doses to 0.163% of the population per day – the average rollout speed across Oceania in 2022 [1]. All antiviral programs presented in the main results are modelled as starting on the 1st of January 2023. We quantified the uncertainty in our projections by conducting 100 runs of the stochastic oral antiviral model for each antiviral strategy and visualising the median and interquartile range in our results. We considered outcomes in 2023 only.

## Results

**Fig. 1:**
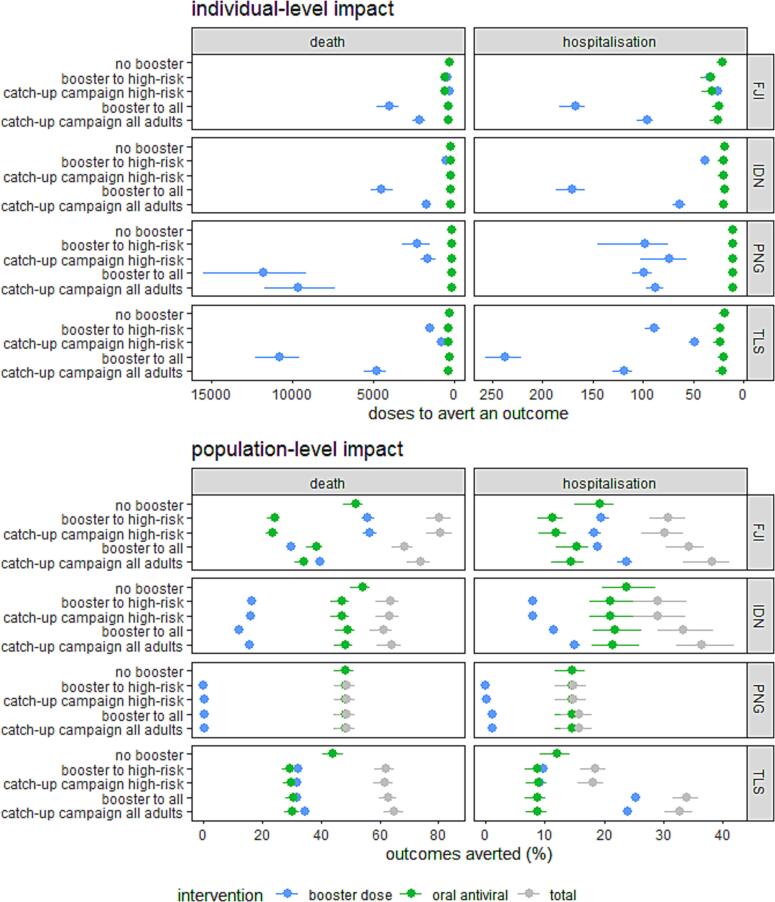
**Comparison between the individual- and population-level impact of booster doses and oral antivirals in 2023.** The individual-level impact refers to the number of booster vaccine doses or courses of oral antivirals required to prevent one hospitalisation or death; the population-level impact refers to the percentage of hospitalisations or deaths averted by a specific strategy. The impact of interventions increases towards the right of each figure pane. These simulations assume that only high-risk adults are eligible for oral antivirals. Five scenarios are provided for booster dose eligibility: no booster doses, high-risk adults eligible, high-risk adults who previously have not received a booster eligible, all adults eligible, and all adults who have not received a booster dose eligible.

**Fig. 2:**
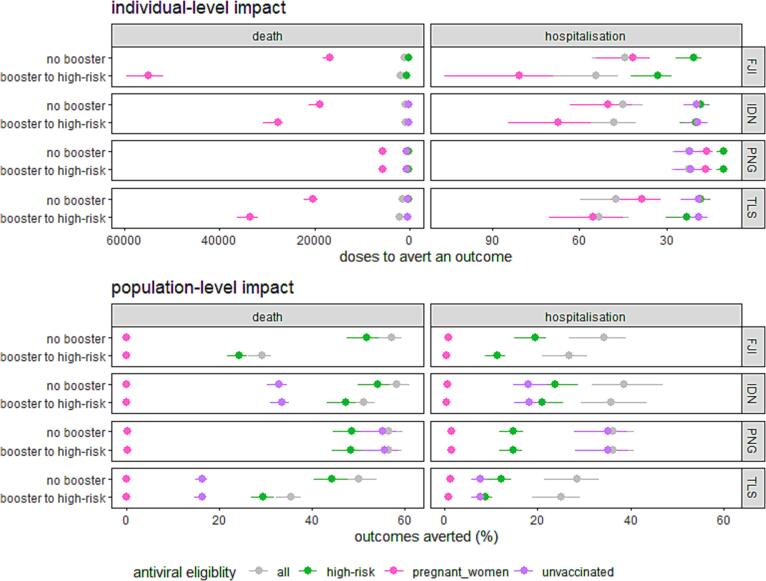
**Comparison between the individual-level impact and population-level impact of different antiviral eligibility criteria in 2023.** The individual-level impact refers to the number of booster vaccine doses or courses of oral antivirals required to prevent one hospitalisation or death; the population-level impact refers to the percentage of hospitalisations or deaths averted by a specific strategy. The impact of the oral antivirals increases towards the right of each figure pane. Outcomes averted are restricted to 2023 and calculated by comparison to the same vaccination scenarios without antivirals. Results for an unvaccinated group are not presented for Fiji due to close to 100% vaccine coverage reported in administrative data.

These results provide projections for the individual- and population-level impact of different eligibility strategies for booster doses (Fig. 1) and oral antiviral programs (Fig. 2) in 2023. A booster dose program paired with oral antivirals to high-risk adults could reduce 49–80% of deaths and 16–38% of hospitalisations in our study settings during 2023. Vaccine acceptance dictated which intervention had the largest population-level impact in a setting. Booster doses had a larger population-level impact in Fiji and Timor-Leste due to high vaccine acceptance (97% and 72% respectively) in adults aged over 60, whereas oral antivirals had a higher population-level impact in Indonesia and Papua New Guinea due to lower (50%) and limited (1%) vaccine acceptance in adults aged over 60.

### Impact of booster doses

Booster doses had the largest individual-level impact when prioritised to high-risk adults or to adults who had not previously received a booster (Fig. 1). At a population-level, providing booster doses to high-risk adults could prevent 20%, 8%, and 10% of COVID-19 related hospitalisations, and 56%, 16%, and 32% of COVID-19 associated deaths in Fiji, Indonesia, and Timor-Leste. By comparison, <1% of deaths or hospitalisations could be averted by booster doses in Papua New Guinea due to low vaccine acceptance. The individual-level impact of booster doses was 2–3 times higher when provided to individuals who had not previously received a booster dose (catch-up campaigns), compared to providing all adults with booster doses in Fiji, Indonesia, and Timor-Leste. Booster programs which only reached the same subset of individuals previously willing to receive booster doses did not have a large impact (Fig. S2.2).

Booster programs which expanded eligibility to all adults without prioritising high-risk adults would have a lower population-level impact on deaths than booster programs vaccinating high-risk adults (Fig. 1). This result in Fiji was due to delays in the vaccination of high-risk adults when eligibility was expanded to all adults due to restricted rollout capacity. Additional results in the Supplementary Materials demonstrate that a multi-staged rollout – vaccinating high-risk adults first and then expanding eligibility to all adults – would be more effective in all settings, increasing the population-level reduction of hospitalisations from 16% to 22%, and deaths from 27% to 52% in Fiji (Fig. S2.1).

### Impact of oral antivirals

Providing oral antivirals to high-risk adults had a meaningful impact on reducing deaths and hospitalisations in all settings and under all vaccination scenarios (Fig. 1). Without booster doses in 2023, an oral antiviral program for high-risk adults could prevent 44–54% of deaths with nirmatrelvir-ritonavir or 10–15% of deaths with molnupiravir across our study settings (Fig. S2.5). Antivirals were more effective in settings with lower vaccine-derived immunity. Half as many antivirals were needed to avert a hospitalisation or death in Papua New Guinea compared to Fiji, Indonesia, or Timor-Leste without booster doses during 2023. In settings with higher vaccine acceptance, the impact of oral antivirals was reduced after the delivery of booster doses. This effect was most noticeable in Fiji, our setting with the highest vaccine coverage, where antivirals were 39% and 60% less effective at an individual level at preventing hospitalisations and deaths after a booster program for high-risk adults (Fig. S2.3).

Antivirals were most effective at preventing severe outcomes in high-risk adults (older adults and adults with comorbidities), followed by unvaccinated adults, before broadening delivery to all adults (Fig. 2). Much of the maximum possible population-level impact of oral antivirals could be achieved by providing oral antivirals to high-risk adults only. For example, in Indonesia, only an additional 4% of deaths (54% to 58%) or 15% of hospitalisations (from 24% to 39%) could be averted by expanding an antiviral program from high-risk adults to all adults (4.1 times more antivirals delivered) without booster doses. The impact of delivering antivirals to unvaccinated adults was inversely related to vaccine acceptance – the highest impact being in Papua New Guinea, Indonesia, and then Timor-Leste. Providing oral antivirals to pregnant women was more effective than their provision to adults the same age (Fig. S2.2), but less effective than their use in older adults and adults with comorbidities (Fig. 2).

### Impact of booster doses and oral antivirals

In general, fewer antivirals were required than booster doses to prevent a hospitalisation or death (Fig. 1). However, the impact of providing a booster dose to high-risk adults was comparable to providing oral antivirals to high-risk adults in Fiji and Indonesia. This comparable effectiveness was attributable to high levels of vaccine coverage providing a short-term reduction in transmission to older adults in Fiji and Timor-Leste (Table S2.1).

## Discussion

Providing booster vaccine doses to high-risk adults remains important as we transition from pandemic to endemic COVID-19. If COVID-19 programs are limited by rollout capacity, catch-up programs providing booster doses to adults who have not yet received their booster dose will have the largest impact. Booster programs had a higher population-level impact than oral antivirals in Fiji and Timor-Leste, two settings with high vaccine coverage. Providing booster doses to high-risk adults could prevent 56% and 32% of deaths in 2023 in Fiji and Timor-Leste respectively. Similar modelling conducted in Hong Kong, Japan, and Vietnam demonstrated that increased primary vaccine coverage (in early 2022) had a higher population-level impact than introducing oral antivirals [24]. Continued public communication alongside booster dose programs are essential to enable widespread uptake and ensure impact. Our sensitivity analysis demonstrated how reaching the same subset of the population who were previously willing to receive a booster dose prevented <10% of deaths and hospitalisations, even in settings with high vaccine coverage.

Our results demonstrate the meaningful impact of oral antivirals provided alongside booster doses in 2023. Even with high vaccine coverage, oral antiviral treatments for high-risk adults could prevent an additional 24–52% of deaths and 12–19% of hospitalisations in Fiji, 47–54% of deaths and 21–24% of hospitalisations in Indonesia, and 30–44% of deaths and 9–12% of hospitalisations in Timor-Leste (range dependent on booster coverage). This result aligns with the benefits of oral antivirals alongside booster programs found in previous modelling by Matrajt and colleagues in Kenya, Mexico, the United States, and Belgium [25].

Oral antivirals demonstrate a large potential to reduce the burden of COVID-19 in settings with low vaccine coverage. In Papua New Guinea, we estimated that up to 49% of deaths and 15% of hospitalisations could be prevented by oral antiviral programs for high-risk adults. This result is particularly impressive given that high-risk adults represent less than 10% of the population in Papua New Guinea. In comparison, less than 1% of deaths or hospitalisations could be prevented by providing booster doses to all adults previously vaccinated. The potential of oral antivirals to have a large impact in settings with low vaccine coverage is supported by previous modelling [25]. This result should be considered when planning COVID-19 mitigation strategies in subnational regions with low vaccine coverage. The usefulness of targetting unvaccinated adults will depend on the acceptability of oral antivirals in this group.

Availability and affordability will determine the true impact of booster doses and oral antivirals in middle-income nations. Our model quantified the impact of booster doses and oral antivirals with sufficient supply and capacity for delivery of these interventions in our study settings. Supply is determined by global production and the purchasing power of nations. Current costs of oral antivirals, between $500-$700 USD per complete schedule in 2022, are unlikely to make them cost-effective in middle-income countries [26]. Delivery depends on healthcare capacity and public willingness to seek care. The burden of testing on the individual or the healthcare system will dictate the achievable coverage of oral antivirals. The WHO acknowledges and recent modelling demonstrates that current levels of testing in low- and middle-income countries are too low to support widespread rollout of oral antivirals [3], [27].

The full benefits or potential adverse effects of introducing an oral COVID-19 antiviral in low-resource settings have not yet been quantified. Clinical trials suggested that patients receiving antivirals experience an accelerated rate of clearance and reduced viral load [12], [28]. Reduced lengths of hospitalisation are likely to have additional benefits related to freeing up health workforce capacity [29]. No significant severe adverse reactions to nirmatrelvir-ritonavir or molnupiravir have been detected in the clinical trials [11], [12]. However, there are concerns that nirmatrelvir-ritonavir may increase the risk of individuals with uncontrolled or undiagnosed HIV developing resistance to protease inhibitors [30]. The hepatotoxicity of ritonavir requires the monitoring of liver function every 3 to 6 months [31], although such long-term use of COVID-19 antivirals is not recommended. Any future implementation of COVID-19 antivirals at a population-level in a low-resource setting should be accompanied by surveillance of adverse events.

Our paper has several notable limitations. First, our results do not capture the full benefits of booster-derived immunity preventing severe outcomes in future years because we focused on outcomes in 2023 only. Our results also do not capture differences in the effectiveness of oral antivirals between settings. Antiviral effectiveness will vary across our study settings due to varying time to access rapid antigen testing and oral antiviral dispensation, and differences in qualities of standard care in our study settings. Further, our model’s fit to reported cases may be over or underestimating infection-derived immunity in our study settings. Varying transmission between the lowest and highest estimates for 2020–2022 did not qualitatively affect the findings of our model for Papua New Guinea during 2023 (Supplementary Material S1.6). Finally, our model does not consider the possibility of antiviral resistance. Antivirals in high-risk adults are unlikely to place sustained selection pressure on the virus since previous models have shown limited effected of antivirals on transmission [32].

## Conclusions

Our results support the continued provision of booster doses to high-risk adults in settings with high vaccine acceptance. Oral antivirals will have a meaningful impact in all settings and the largest impact in settings with low vaccine coverage. The current availability and affordability of oral antivirals is likely to limit their use in middle-income countries. Already, supplies of humanitarian funded oral antivirals are not meeting demands from low- and middle-income countries [33]. Future work should consider cost-effectiveness and the threshold at which self-financing of COVID-19 oral antivirals would be viable for middle-income countries in South-East Asia and the Pacific.

## Contributors

All authors attest they meet the ICMJE criteria for authorship. All authors designed the study. KL and SLM informed the study settings’ priorities for the evaluation of interventions against COVID-19. GMB and KG designed the oral antiviral model. GMB undertook the literature review, developed the code, and wrote the original draft. All authors assisted in the interpreting of the results and review of the manuscript.

## Funding

This research did not receive any specific grant from funding agencies in the public, commercial, or not-for-profit sectors. GMB was supported by the Australian Government Research Training Program stipend during this study. This scholarship did not influence the design of the study, writing of the manuscript, or decision to submit for publication.

## Declaration of Competing Interest

The authors declare that they have no known competing financial interests or personal relationships that could have appeared to influence the work reported in this paper.

## Data Availability

All data was collated from publicly available sources. Our model code and inputs can be viewed on GitHub: https://github.com/gizembilgin/indoPacific_COVID19_model.

## References

[1] Mathieu E., Ritchie H., Ortiz-Ospina E., Roser M., Hasell J., Appel C. (2021). A global database of COVID-19 vaccinations. Nat Hum Behav.

[2] World Health Organization (2022).

[3] World Health Organization. Therapeutics and COVID-19: living guideline, 13 January 2023. Geneva: World Health Organization; 2023.

[4] Bilgin GM, Lokuge K, Jabbie E, Munira SL, Glass K. COVID-19 vaccination strategies in settings with limited rollout capacity: a mathematical modelling case study in Sierra Leone. [preprint] avaliable at Research Square. 2023. https://doi.org/10.21203/rs.3.rs-2460525/v1. 10.1186/s12889-023-17374-0 PMC10712073 38082260

[5] The World Bank. World Bank Open Data (2022). https://data.worldbank.org/. Accessed 22/11/2022.

[6] United Nations Department of Economic and Social Affairs Population Division. World Population Prospects 2019. Online Edition.

[7] Clark A., Jit M., Warren-Gash C., Guthrie B., Wang H.H.X., Mercer S.W. (2020). Global, regional, and national estimates of the population at increased risk of severe COVID-19 due to underlying health conditions in 2020: a modelling study. Lancet Glob Health.

[8] Dong E., Du H., Gardner L. (2020). An interactive web-based dashboard to track COVID-19 in real time. Lancet Infect Dis.

[9] Feikin D.R., Abu-Raddad L.J., Andrews N., Davies M.A., Higdon M.M., Orenstein W.A. (2022). Assessing vaccine effectiveness against severe COVID-19 disease caused by omicron variant. Report from a meeting of the World Health Organization. Vaccine.

[10] World Health Organization. Therapeutics and COVID-19: living guideline, 16 September 2022. Geneva: World Health Organization; 2022. 35917393

[11] Jayk Bernal A., Gomes da Silva M.M., Musungaie D.B., Kovalchuk E., Gonzalez A., Delos Reyes V. (2022). Molnupiravir for Oral Treatment of Covid-19 in Nonhospitalized Patients. N Engl J Med.

[12] Hammond J., Leister-Tebbe H., Gardner A., Abreu P., Bao W., Wisemandle W. (2022). Oral Nirmatrelvir for High-Risk, Nonhospitalized Adults with Covid-19. N Engl J Med.

[13] Siberry G.K., Mofenson L.M., Calmy A., Reddy U.M., Abrams E.J. (2022). Use of Ritonavir-boosted Nirmatrelvir in Pregnancy. Clin Infect Dis.

[14] The University of Liverpool. Liverpool COVID-19 Drug Interactions (2023). https://www.covid19-druginteractions.org/checker. Accessed 09/03/2023.

[15] National Institutes of Health. Drug-Drug Interactions Between Ritonavir-Boosted Nirmatrelvir (Paxlovid) and Concomitant Medications (2023). https://www.covid19treatmentguidelines.nih.gov/therapies/antivirals-including-antibody-products/ritonavir-boosted-nirmatrelvir--paxlovid-/paxlovid-drug-drug-interactions/. Accessed 10/05/2023.

[16] Stolbrink M., Thomson H., Hadfield R.M., Ozoh O.B., Nantanda R., Jayasooriya S. (2022). The availability, cost, and affordability of essential medicines for asthma and COPD in low-income and middle-income countries: a systematic review. Lancet Glob Health.

[17] Husain M.J., Datta B.K., Kostova D., Joseph K.T., Asma S., Richter P. (2020). Access to Cardiovascular Disease and Hypertension Medicines in Developing Countries: An Analysis of Essential Medicine Lists, Price, Availability, and Affordability. J Am Heart Assoc.

[18] Wang H, Sun Q, Vitry A, Nguyen TA. Availability, Price, and Affordability of Selected Essential Medicines for Chronic Diseases in 11 Countries of the Asia Pacific Region A Secondary Analysis. Asia Pac J Publ Health. 2017;29:268–77. 10.1177/1010539517700472 28397532

[19] Menkir T.F., Donnelly C.A. (2022). The impact of repeated rapid test strategies on the effectiveness of at-home antiviral treatments for SARS-CoV-2. Nat Commun.

[20] Zheng Q., Ma P., Wang M., Cheng Y., Zhou M., Ye L. (2022). Efficacy and safety of Paxlovid for COVID-19:a meta-analysis. J Infect.

[21] Najjar-Debbiny R., Gronich N., Weber G., Khoury J., Amar M., Stein N. (2022). Effectiveness of Paxlovid in Reducing Severe COVID-19 and Mortality in High Risk Patients. Clin Infect Dis.

[22] Wong C.K.H., Au I.C.H., Lau K.T.K., Lau E.H.Y., Cowling B.J., Leung G.M. (2022). Real-world effectiveness of molnupiravir and nirmatrelvir plus ritonavir against mortality, hospitalisation, and in-hospital outcomes among community-dwelling, ambulatory patients with confirmed SARS-CoV-2 infection during the omicron wave in Hong Kong: an observational study. Lancet.

[23] Merck Sharp & Dohme Corporation. Merck and Ridgeback Biotherapeutics Provide Update on Results from MOVe-OUT Study of Molnupiravir, an Investigational Oral Antiviral Medicine, in At Risk Adults With Mild-to-Moderate COVID-19 (2021). https://www.merck.com/news/merck-and-ridgeback-biotherapeutics-provide-update-on-results-from-move-out-study-of-molnupiravir-an-investigational-oral-antiviral-medicine-in-at-risk-adults-with-mild-to-moderate-covid-19/. Accessed 13/09/2022.

[24] Leung K., Jit M., Leung G.M., Wu J.T. (2022). The allocation of COVID-19 vaccines and antivirals against emerging SARS-CoV-2 variants of concern in East Asia and Pacific region: A modelling study. Lancet Reg Health West Pac.

[25] Matrajt L., Brown E.R., Cohen M.S., Dimitrov D., Janes H. (2022). Could widespread use of antiviral treatment curb the COVID-19 pandemic? A modeling study. BMC Infect Dis.

[26] Insitute for Clinical and Economic Review. Special Assessment of Outpatient Treatments for COVID-19:Final Evidence Report and Meeting Summary. 2022.

[27] Han AX, Hannay E, Carmona S, Rodriguez B, Nichols BE, Russell CA. Estimating the potential need and impact of SARS-CoV-2 test-and-treat programs with oral antivirals in low-and-middle-income countries. medRxiv [preprint]. 2022:2022.10.05.22280727. https://doi.org/10.1101/2022.10.05.22280727.

[28] Fischer W.A., Eron J.J., Holman W., Cohen M.S., Fang L., Szewczyk L.J. (2022). A phase 2a clinical trial of molnupiravir in patients with COVID-19 shows accelerated SARS-CoV-2 RNA clearance and elimination of infectious virus. Sci Transl Med.

[29] Johnson M.G., Puenpatom A., Moncada P.A., Burgess L., Duke E.R., Ohmagari N. (2022). Effect of Molnupiravir on Biomarkers, Respiratory Interventions, and Medical Services in COVID-19: A Randomized, Placebo-Controlled Trial. Ann Intern Med.

[30] UK Medicines & Healthcare products Regulatory Agency. Decision: Summary of Product Characteristics for Paxlovid. 2022.

[31] Talha B, Dhamoon AS. Ritonavir. StatPearls. Treasure Island (FL) 2022.

[32] Ngonghala C.N., Taboe H.B., Safdar S., Gumel A.B. (2023). Unraveling the dynamics of the Omicron and Delta variants of the 2019 coronavirus in the presence of vaccination, mask usage, and antiviral treatment. App Math Model.

[33] Ledford H. (2022). Donated COVID drugs start flowing to poor nations - but can't meet demand. Nature.

