# Supplementary material for: Assessing the potential impact of COVID-19 booster doses and oral antivirals: A mathematical modelling study of selected middle-income countries in the Indo-Pacific
Source: Vaccine X. 2023 Sep 9;15:100386. doi: 10.1016/j.jvacx.2023.100386 (PMC10506093; doi:10.1016/j.jvacx.2023.100386)
Supplement: Supplementary data 1 [file mmc1.docx]

**Assessing the potential impact of COVID-19 booster doses and oral antivirals: a mathematical modelling study of selected middle-income countries in the Indo-Pacific**

Supplementary Material

Table of Contents

[S1. Additional Methods 2](#_Toc142465767)

[**S1.1 Model structure** 2](#_Toc142465768)

[**S1.2 Reconstructing vaccination history** 3](#_Toc142465769)

[**S1.3 Vaccine effectiveness** 3](#_Toc142465770)

[**S1.3.1 First booster** 3](#_Toc142465771)

[**S1.3.2 Second booster** 5](#_Toc142465772)

[**S1.4 Introduction of new variants** 5](#_Toc142465773)

[**S1.5 Fit** 6](#_Toc142465774)

[**S1.6 Ensemble fit – special case for Papua New Guinea** 8](#_Toc142465775)

[**S1.7 Visualisation of differences in prevalence of comorbidities, age-structure, and vaccine coverage between study settings** 10](#_Toc142465776)

[S2. Additional Results 11](#_Toc142465777)

[**S2.1 Booster dose impact** 11](#_Toc142465778)

[**S2.1.1 ‘Cocooning’ of older adults by high vaccine coverage** 11](#_Toc142465779)

[**S2.1.2 Booster dose campaigns with multi-stage prioritisation** 12](#_Toc142465780)

[**S2.1.3 Varying acceptance of future booster doses** 13](#_Toc142465781)

[**S2.2 Antiviral impact** 14](#_Toc142465782)

[**S2.2.1 Impact of antivirals relative to timing of booster programs** 14](#_Toc142465783)

[**S2.2.2 Use of an oral antiviral for pregnant women** 15](#_Toc142465784)

[**S2.2.3 Use of an oral antiviral with lower effectiveness (molnupiravir)** 16](#_Toc142465785)

[Reference List 17](#_Toc142465786)

# S1. Additional Methods

## **S1.1 Model structure**

This section provides a summary of the COVID-19 transmission model underlying this paper. Further detail on the design and execution of the model can be found in the previous paper and Supplementary Material (in preprint [1]).

The COVID-19 transmission model used a Susceptible-Exposed-Infected-Recovered (SEIR) structure (Figure S1.1). Our model focused on exploring age-, dose- and risk-specific prioritisation of booster doses and oral antivirals, hence, was subdivided by age, vaccine-derived immunity by dose and type, and risk group. Both vaccine- and infection-derived immunity were updated during a daily time step.


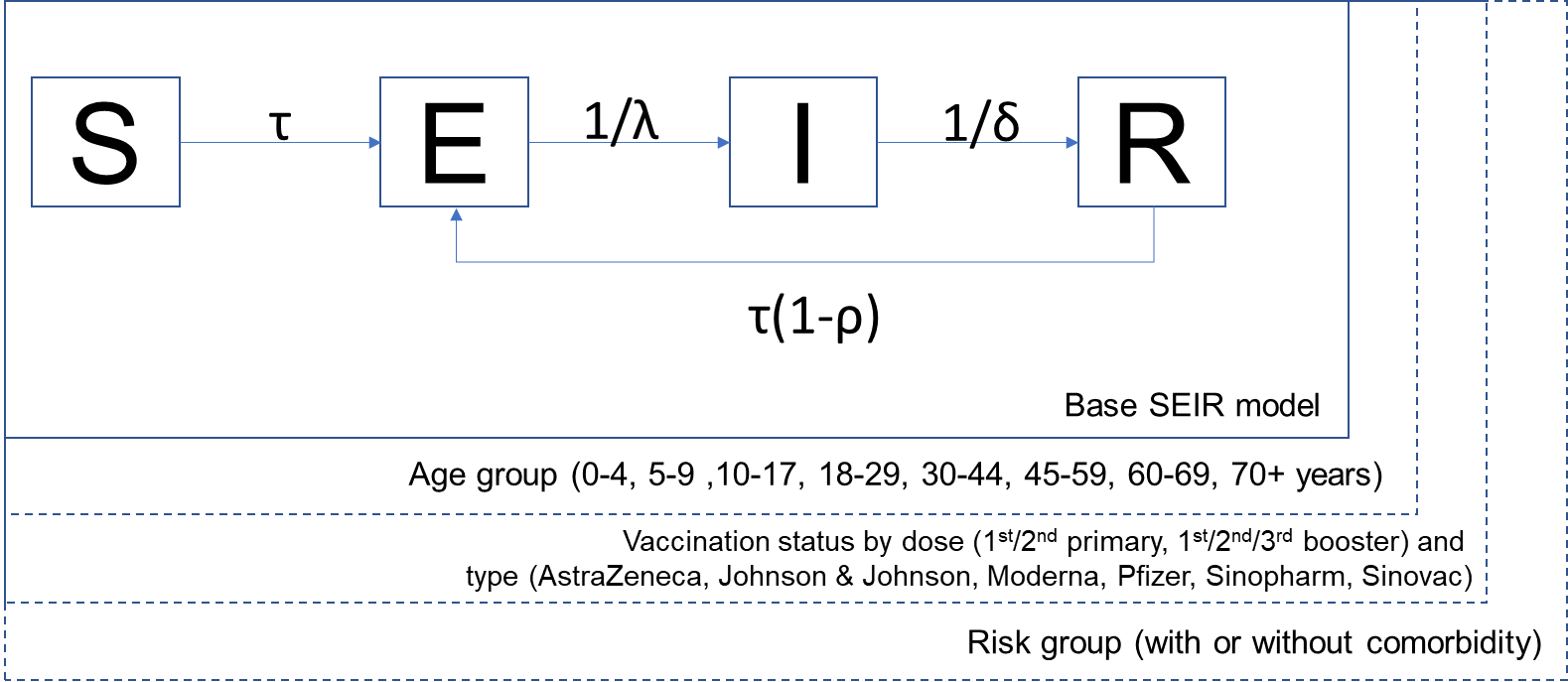


**Figure S1.1** Schematic for model structure including the progression of individuals between Susceptible (S), Exposed (E), Infected (I) and Recovered (R) classes. The parameter $\tau$ represents the force of transmission, λ the latent period (3.71 days delta [2], 2.22 days omicron [3]), δ the infectious period (10.9 days delta, 9.87 days omicron [4]), and ρ the effectiveness of infection-derived immunity.

Estimates for all basic transmission parameters were kept constant between settings. Differences in transmission dynamics arose from setting-specific differences in age-structure [5], contact patterns [6], time-varying effectiveness of non-pharmaceutical interventions [7], and vaccine coverage by type and dose (see S1.2). Differences in projections of severe outcomes arose from setting-specific differences in the prevalence of comorbidities [8] and severity of infection influenced by access to care [9-11]. Section S1.7 of this Supplementary Material contains visualisations for some of these key differences.

For each age group $i$, transmission ($\tau$) was modelled by:

$$\tau\left( i \right)= \beta*u_{i}*(1-NPI)*\sum_{j} C_{i,j}*\frac{I\left( j \right)}{N\left( j \right)}*\left( \iota\left( 1-\gamma\left( j \right) \right)+\gamma(j) \right).$$

Where $C_{i,j}$ represents their daily contact with other age groups, $\frac{I\left( j \right)}{N\left( j \right)}$ the proportion of their contacts who are infected, $\gamma$ the proportion of infected contacts who are symptomatic [12], and $\iota$ is the reduced infectiousness of asymptomatic cases (0.50 as per [13-15]). Transmission was further adjusted by age-specific susceptibility to infection ($u_{i})$ [12], the effectiveness of non-pharmaceutical interventions $(1-NPI)$, and a scaling factor to fit transmission to the basic reproduction number of the circulating strain ($\beta)$.

For vaccinated individuals, transmission was also modulated by vaccine effectiveness by time since vaccination:

$$\tau\left( i \right)= \tau\left( i \right)*\left( {1- VE}_{dose, vaccine type}[days] \right).$$

For individuals in the recovered class, transmission was also modulated by the effectiveness of infection-derived immunity by time since previous infection:

$$\tau\left( i \right)= \tau\left( i \right)*\left( 1-\rho[days] \right).$$

## **S1.2 Reconstructing vaccination history**

Our model used daily time-steps to accurately reflect the vaccination history of our study settings. We derived the number of doses delivered on each day using data collated by Our World in Data [16]. When distributing doses across age- and risk-groups, we first referred to official government announcements (Indonesia [17, 18], Fiji [19-23], Papua New Guinea [24, 25]), national level WHO situational reports (Timor-Leste [26]), and then to the Oxford COVID-19 Government Response Tracker [7]. Where available, we used government reporting or national level WHO situational reports on the vaccine type of these doses delivered. Otherwise, we inferred the vaccine type of doses delivered using monthly national vaccine procurement data from the UNICEF’s COVID-19 Market Dashboard [27]. We included vaccine types that covered at least 5% of the population, otherwise including doses with the closest available vaccine (e.g., Pfizer with Moderna, or Covishield with AstraZeneca). We projected the continuation of existing vaccine programs using the vaccine type used in majority for each age group over the last three months of 2022 (Fiji: Moderna, Indonesia: Pfizer, Papua New Guinea: Johnson & Johnson, Timor-Leste: Pfizer).

## **S1.3 Vaccine effectiveness**

Our model includes vaccine effectiveness (VE) against infection, severe disease, and death dependent on time since vaccination. We provide more detailed methods on the estimation of first and second dose effectiveness in the Supplementary Material of the preprint of our previous paper [1]. Briefly, we estimate VE by vaccine type, dose, outcome, and circulating strain using estimates in the living systematic review jointly conducted by the International Vaccine Access Center and World Health Organisation [28]. To these point estimates, we applied an exponential distribution of waning immunity fit to data by Andrews, Stowe [29] for VE against infection, and Cerqueira-Silva, de Araujo Oliveira [30], [31] for VE against severe outcomes.

### **S1.3.1 First booster**

We estimated the effectiveness of a booster dose by calculating a weighted average across estimates of heterologous combinations as expected in our study settings. That is, to estimate the effectiveness of Pfizer booster doses we considered VE estimates from studies where Pfizer boosters were delivered to individuals who had received AstraZeneca, Sinopharm, or Pfizer primary schedules.

**Table S1.1** Vaccine types for primary and booster doses in our study settings

|  | **Primary vaccines** | **Booster vaccines** |
| --- | --- | --- |
| Fiji | AstraZeneca, Moderna, Pfizer | Pfizer, Moderna |
| Indonesia | AstraZeneca, Moderna, Pfizer, Sinovac | AstraZeneca, Pfizer, Sinovac |
| Papua New Guinea | AstraZeneca, Johnson & Johnson, Sinopharm | Johnson & Johnson, AstraZeneca |
| Timor-Leste | AstraZeneca, Pfizer, Sinovac | AstraZeneca, Pfizer, Sinovac |

Where an estimate of VE for a booster dose + primary schedule combination was not available we imputed an estimate from the most relevant available estimate in the following order: outcome (heterologous and homologous combinations), strain (heterologous and homologous combinations), dose (homologous combinations only). First, we imputed an estimate from VE against a similar outcome for the same primary-booster combination. VE against death and VE against severe disease aligned broadly (ratio 0.96, σ 0.07). The ratio of effectiveness between VE against any infection and VE against symptomatic infection was 0.74 (σ 0.11). The difference in VE against delta and omicron variants reduced with severity: any infection (ratio 0.53, σ 0.04), severe disease (ratio 0.91, σ 0.04), death (ratio 0.97, σ 0.05). The ratio of VE between two and three doses (homologous combinations) was greater for Omicron (ratio 1.28, σ 0.17) than Delta (ratio 1.03, σ 0.04). Where an estimate could not be reasonably imputed, we used an average across the same vaccine medium: in-activated vaccines (e.g., Sinovac, Sinopharm), viral-vector vaccines (e.g., AstraZeneca and Johnson & Johnson), or mRNA vaccines (e.g., Pfizer and Moderna).


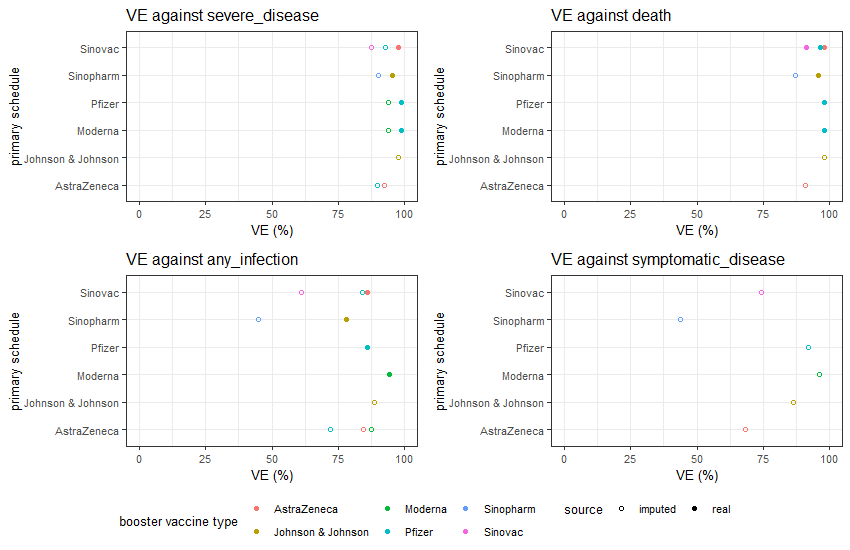
 **Figure S1.2 Booster dose effectiveness against Delta by varying primary schedule [32-37] as selected from [28].**


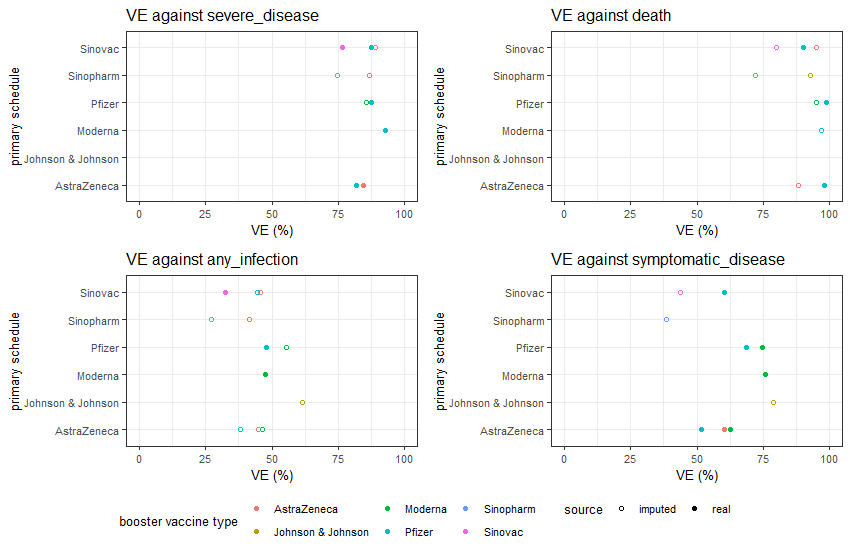


**Figure S1.3 Booster dose effectiveness against Omicron by varying primary schedule [29, 30, 37-45] as selected from [28].**

### **S1.3.2 Second booster**

We used estimates of the effectiveness of a second booster dose where available, and otherwise assumed that second booster doses were at least as effective as first booster doses. Overall, there were limited data on the absolute effectiveness of a second booster dose in the general population [28]. Most existing studies reported the relative effectiveness of a second booster dose compared to individuals who have received a first booster. Other available studies reported the effectiveness of a second booster dose in immunocompromised cohorts.

Similarly, there was little data on the waning of immunity derived from a second booster dose. The December 2022 update to the World Health Organization and International Vaccine Access Centre’s living systematic review of vaccine effectiveness [28] identified only one study which estimated the waning of the absolute effectiveness of a second booster dose in long term care facility patients aged over 60 [46], and two studies estimating the waning of the relative effectiveness of a second booster compared to a first booster in health care workers [47], and individuals aged over 80 [48]. Given the absence of data, we assumed equal waning between a first and second booster dose.

## **S1.4 Introduction of new variants**

We estimated the speed at which new variants outcompeted previously circulating variants using Oceania samples reported to GISAID (Figure S1.4) [49]. We utilised Oceania samples for all study settings since Asian samples reported to GISAID were overrepresented by larger nations including China. We assumed all pre-Omicron strains followed the same speed of introduction as Delta, and all Omicron strains had equal speeds of introduction.


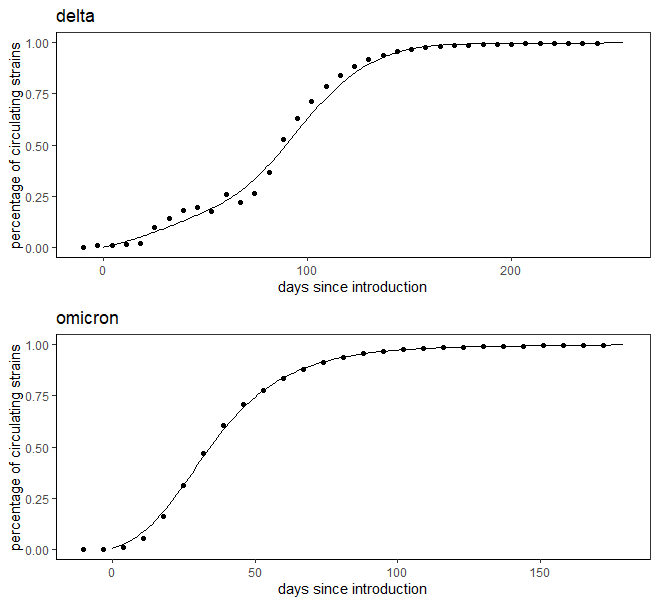


**Figure S1.4 Model estimates of the speed of introduction of new variants fit to sequenced Oceania reported to GISAID [49].** Points represent weekly reported percentages, and the line represents the fitted curve.

## **S1.5 Fit**

Our model was fitted to previous transmission to estimate the prevalence of infection-derived immunity $\left( \rho\right)$in our study settings. We fitted our model to daily reported COVID-19 using the *DEoptim* package [50]. We conducted fitting after including our best estimate of vaccine-derived immunity in our settings and using best available evidence for basic disease parameters. We fitted three parameters: introduction dates for new variants, the under reporting of cases, and transmission potential per wave. We fitted each previous wave of COVID-19 individually using the fit statistic:

$$F= \sum_{i=1}^{n} {(reported cases_{i}*under reporting-{predicted cases}_{i})}^{2}$$

As an exception for Indonesia, we used a known point of seroprevalence in March 2021 as the start point of the fit. There were no clear waves prior to delta in Indonesia to which the model could be fit. We used age-specific seroprevalence estimates published in the Indonesian National Public Health Journal [51], which aligned well with other available literature estimating seroprevalence in Indonesia during early 2021 [52-54].

As an exception for Timor-Leste, we fitted to reported cases using an additional modification factor to increase the speed of the introduction of delta. The delta wave in Timor-Leste occurred almost immediately after the first wave. Daily reported cases suggested that delta was introduced faster into Timor-Leste than in neighbouring settings. Hence, we included an additional parameter to truncate the speed of introduction of delta in Timor-Leste which was estimated to be 0.15.

The values of the fitted parameters are provided in Table S1.2. A common trend between study settings was an estimated decrease in the proportion of cases reported (i.e., an increase in under reporting) over time. This increase in the under reporting of cases over time may be explained by a reduction in the proportion of cases testing, a shift from laboratory-testing to rapid-antigen testing, and/or an increase in the prevalence of asymptomatic disease, especially with increased vaccine coverage. Variation in the transmission potential modifier may be explained by differences in the effectiveness of non-pharmaceutical interventions. For example, the higher underreporting in the first wave of COVID-19 compared to the second wave in Timor-Leste can likely be explained by increased crowding due to the flooding caused by Cyclone Seroja [55].

**Table S1.2** Summary of fit to Indonesia, Fiji, and Timor-Leste

| Setting | Wave | Strain | Fitted introduction date | Fitted transmission potential modifier | Fitted underreporting |
| --- | --- | --- | --- | --- | --- |
| Indonesia | 2^nd^ | Delta | 12/04/2021 | 2.0 | 66 |
|  | 3^rd^ | Omicron | 27/12/2021 | 3.9 | 584 |
| Fiji | 1^st^ | Delta | 31/05/2021 | 3.0 | 16 |
|  | 2^nd^ | Omicron | 17/10/2021 | 5.5 | 266 |
|  | 3^rd^ | Omicron | 12/05/2022 | 2.2 | 847 |
| Timor-Leste | 1^st^ | Wild type | 25/02/2021 | 2.5 | 125 |
|  | 2^nd^ | Delta | 20/07/2021 | 5.7 | 109 |
|  | 3^rd^ | Omicron | 01/01/2022 | 0.5 | 321 |


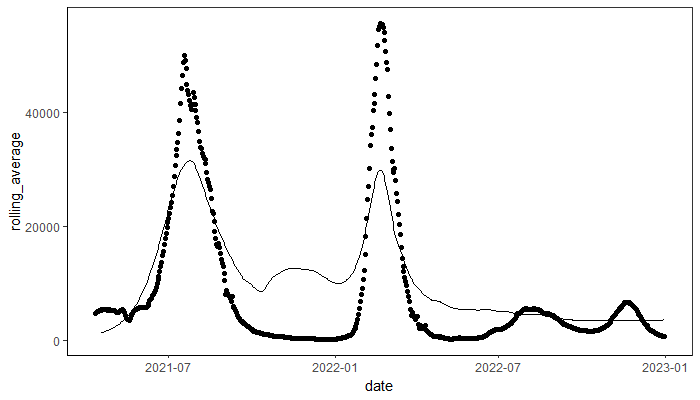


**Figure S1.5 Fit of transmission model to Indonesia.** Points represent daily reported cases, and the line represents model projections.


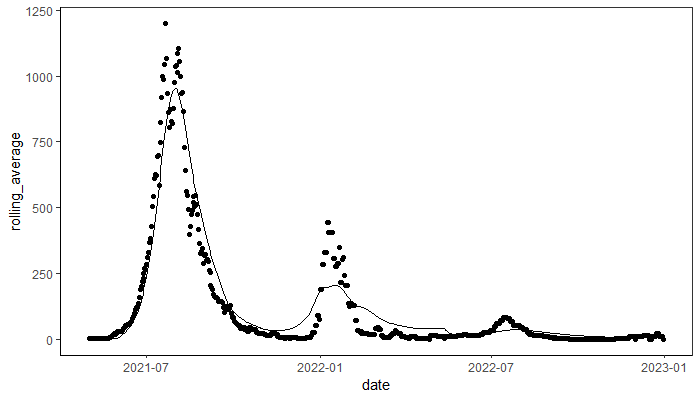


**Figure S1.6 Fit of transmission model to Fiji.** Points represent daily reported cases, and the line represents model projections.

**
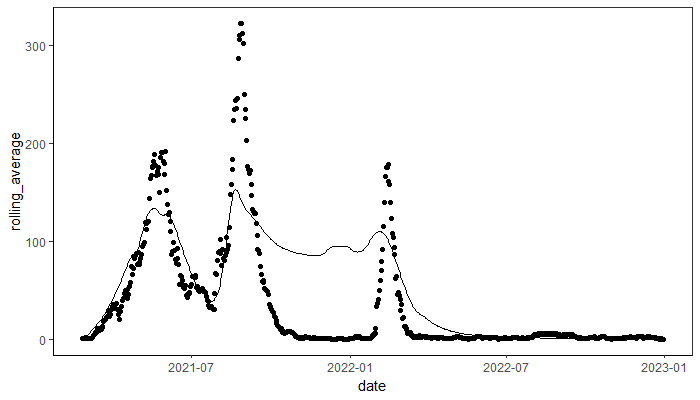
Figure S1.7 Fit of transmission model to Timor-Leste.** Points represent daily reported cases, and the line represents model projections.

## **S1.6 Ensemble fit – special case for Papua New Guinea**

The process of fitting our model to daily reported COVID-19 cases depended on daily reported case numbers being representative of transmission in our study settings. This dependency did not hold for Papua New Guinea due to challenges with health surveillance. Testing capacity has been limited in Papua New Guinea due to a shortage of COVID-19 tests and restricted health workforce capacity [56]. Alternative seroprevalence data was also not available [57].

So, instead of fitting to daily reported cases, we conducted modelling with two possible scenarios of previous transmission in Papua New Guinea, representing the possible range of infection-derived immunity at the start of our simulations (‘low’ β =1.4, ‘high’ β =1.8). We selected these two β modifiers after visualising a range of possible beta values (Figure S1.8), and their effect on the age distribution of infection-derived immunity (Figure S1.9). The difference in the two beta modifier estimates corresponded with 76.6% and 80.6% of population having infection derived immunity at the start of our simulations in 2023.

Antiviral model results did not differ greatly using these two beta modifier estimates, hence, results presented in the main paper are for β =1.4 only. All qualitative decisions surrounding the allocation of booster doses and antivirals remained the same. Booster doses and oral antivirals had a slightly larger impact (<1% difference) in a setting with higher transmission (high beta).


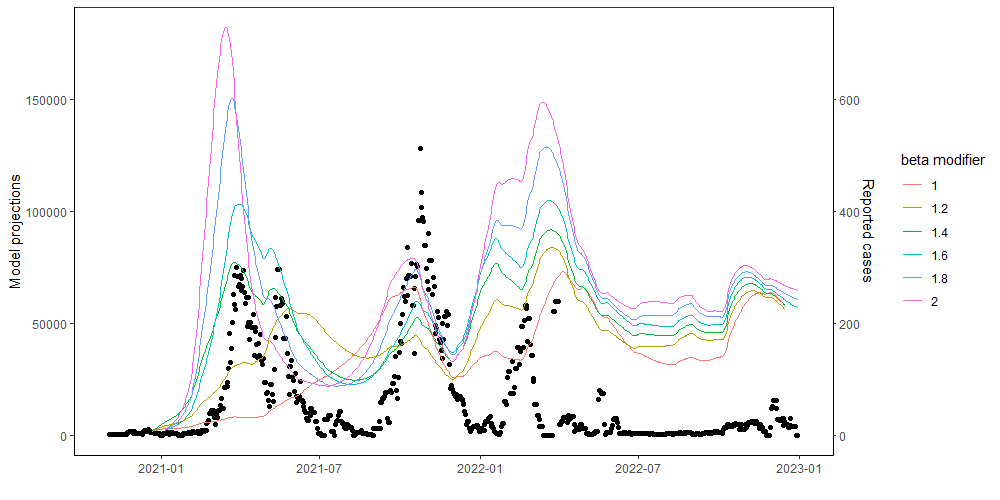
**Figure S1.8 Range of possible beta modifier values for Papua New Guinea.**


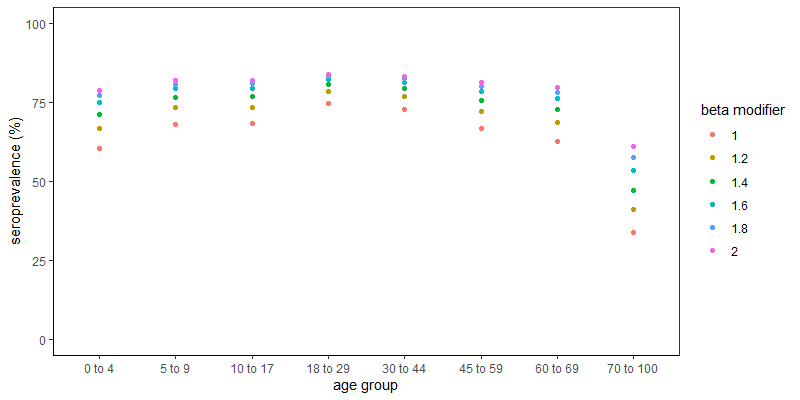


**Figure S1.9 Effect of possible beta modifier values on the age distribution of seroprevalence at the start point of simulations (01/01/2023) for Papua New Guinea.**

## **S1.7 Visualisation of differences in prevalence of comorbidities, age-structure, and vaccine coverage between study settings**

Here we visualise the differences in the age-specific prevalence of comorbidities (Figure S1.10), size of the ‘high-risk’ group (Figure S1.11), and vaccine coverage at the start of 2023 (Figure S1.12) between our study settings.


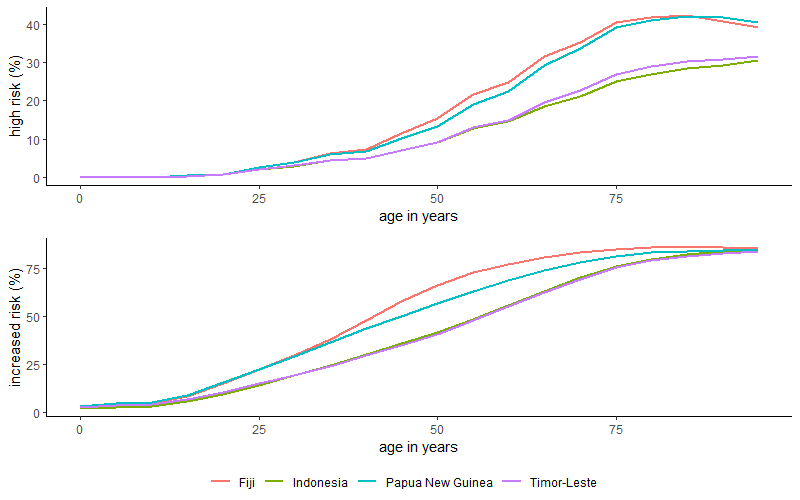


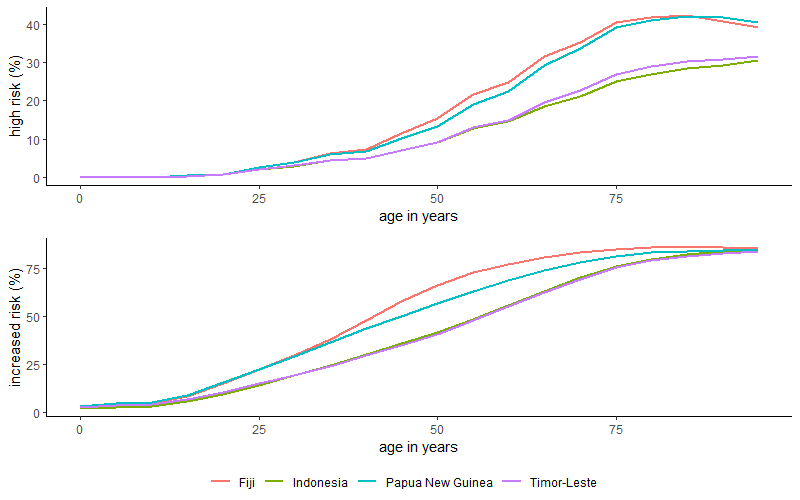


**Figure S1.10 Age-specific proportion of individuals at high-risk of severe outcomes associated with COVID-19 [8].**


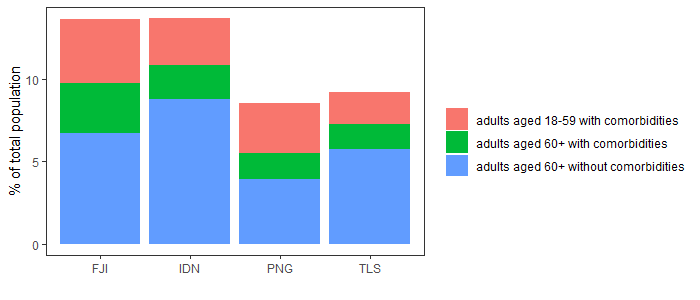


**Figure S1.11 Comparison of the setting-specific proportion of the population identified as high-risk due to age [5], comorbidities [8], or both.**


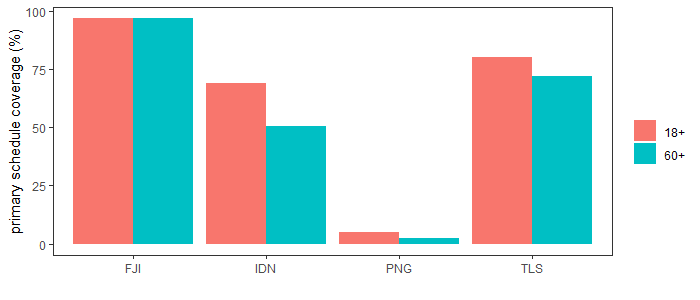


**Figure S1.12 Comparison of the coverage of a primary schedule of COVID-19 vaccines across settings on the 01/01/2023 [58].**

# S2. Additional Results

The results presented in the main paper address the following research questions:

- Would additional booster doses and/or oral antivirals have a meaningful impact on reducing deaths and hospitalisations Fiji, Indonesia, Timor-Leste, and Papua New Guinea during 2023?
- Which setting characteristics influence the individual- and population-level impact of booster doses? Do the same characteristics influence the impact of oral antivirals?
- How do the individual- and population-level impact of providing booster doses or oral antivirals to high-risk adults compare to providing these interventions to all adults?
- What would be the benefit of catch-up campaigns targeting individuals who were willing to receive their primary schedule but have not yet received a booster dose?
- What would be the benefit of targeting unvaccinated individuals to receive oral antivirals?

This Supplementary Material contains results answering the following additional research questions:

- How will ongoing vaccine acceptance affect the impact of booster programs? (S2.1.2)
- To what extent would the impact of booster programs be improved by multi-stage prioritisation? (S2.1.3)
- To what extent would a booster program reduce the impact of an oral antiviral program? (S2.2.1)
- What would be the benefit of prioritising pregnant women for oral antivirals? (S2.2.2)
- Would oral antivirals with lower effectiveness still have a meaningful impact in our study settings? (S2.2.3)

## **S2.1 Booster dose impact**

Sensitivity analysis described in S2.1.2 and S2.1.3 do not include uncertainty in the effectiveness of booster doses due to limitations in available computational resources.

### **S2.1.1 ‘Cocooning’ of older adults by high vaccine coverage**

High vaccine acceptance further improved the benefits of booster doses to older adults by reducing transmission to them from their frequent contacts – other older adults (Table S2.1). This contributed to a higher impact of booster per dose in Fiji, Indonesia, and Timor-Leste compared to in Papua New Guinea.

**Table S2.1** Comparison in the reduction of transmission in the population and to older adults by vaccination scenario

| Setting | Vaccination Scenario | Reduction in transmission in the population (%) | Reduction in transmission in individuals aged over 60 (%) |
| --- | --- | --- | --- |
| Fiji | Booster to high-risk adults | 1.3 | 7.8 |
| Fiji | Booster to all adults | 5.5 | 7.9 |
| Indonesia | Booster to high-risk adults | 0.6 | 3.2 |
| Indonesia | Booster to all adults | 5.6 | 5.4 |
| Papua New Guinea | Booster to high-risk adults | 0.0 | 0.2 |
| Papua New Guinea | Booster to all adults | 0.3 | 0.2 |
| Timor-Leste | Booster to high-risk adults | 1.0 | 11.6 |
| Timor-Leste | Booster to all adults | 18.1 | 23.0 |

### **S2.1.2 Booster dose campaigns with multi-stage prioritisation**

In settings with limited rollout capacity, expanding vaccine eligibility to a larger group slows the delivery of doses to previously prioritised groups. In our main results, we observed that expanding eligibility from high-risk adults to all adults reduced the proportion of deaths prevented by booster dose, most notably in Fiji. Here, we present simulations where high-risk adults were prioritised before the broadening of eligibility to all adults. Figure S2.1 demonstrates how the population-level impact of booster doses is improved by a multi-staged program which prioritises the delivery of booster doses to high-risk adults first.

**
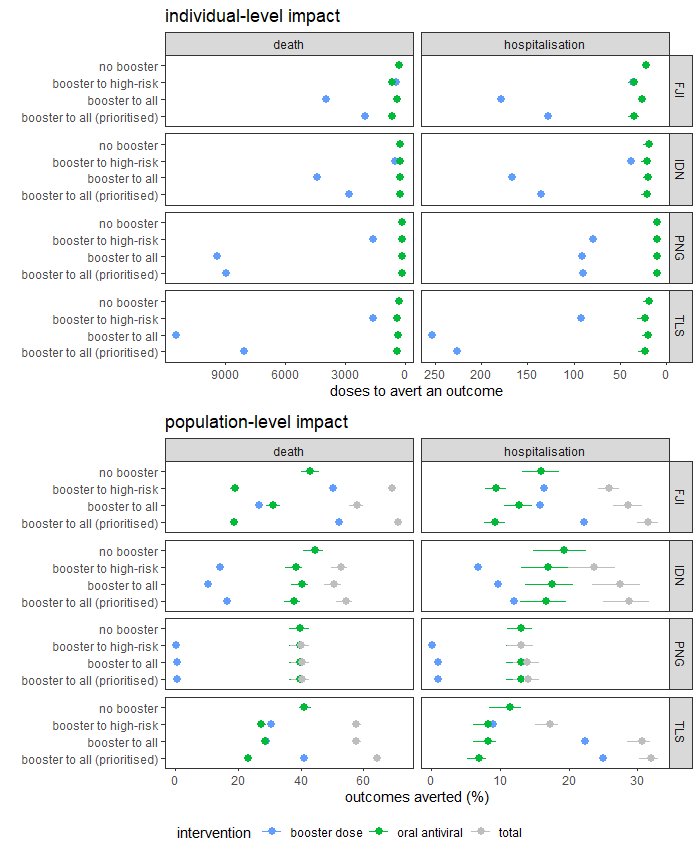
**

**Figure S2.1 Comparison between the impact of high-risk adults receiving boosters, all adults receiving boosters, and a prioritised strategy where high-risk adults receive boosters before broadening eligibility to all adults.**

### **S2.1.3 Varying acceptance of future booster doses**

Ongoing vaccine acceptance is uncertain. Individuals who were previously willing to be vaccinated may not necessarily be willing to receive further doses. In the main paper, we assumed that all individuals who had completed their primary schedule would be willing to receive a booster dose in 2023. Here, we present simulations where only individuals who had previously received a booster dose are willing to receive an additional booster dose in 2023. These results demonstrate that booster dose campaigns will have a lower impact if only the same subset of the population continues to accept booster doses.

**
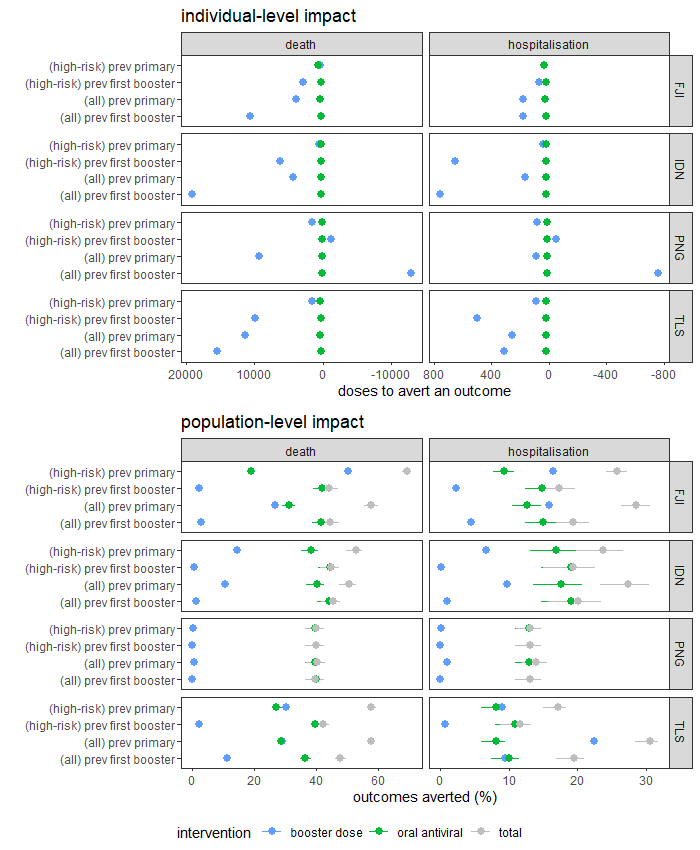
Figure S2.2 Comparison of the effect of booster doses where individuals who previously completed their primary schedule or individuals who previously received a booster dose are willing to receive a booster in 2023.**

## **S2.2 Antiviral impact**

### **S2.2.1 Impact of antivirals relative to timing of booster programs**

These additional results illustrate how the impact of an antiviral program is dependent on its timing relative to a booster dose program. Oral antivirals have a larger impact when rolled out prior to a booster dose, except in settings with low uptake of booster doses such as Papua New Guinea.


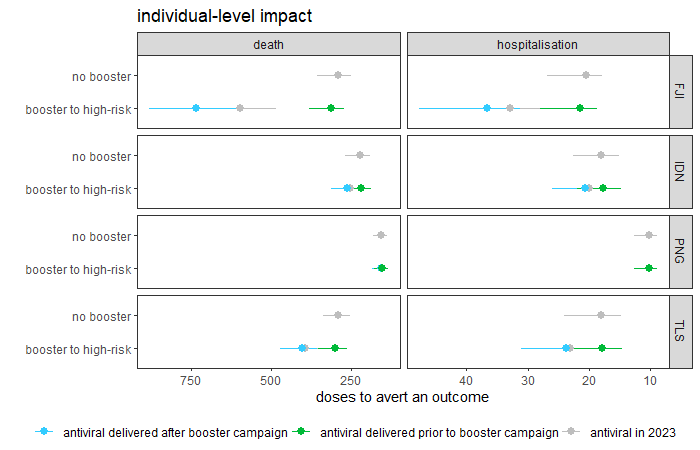


**Figure S2.3 Comparison of the impact of oral antivirals provided to high-risk adults prior to or after the rollout of a booster program starting in March 2023.**

### **S2.2.2 Use of an oral antiviral for pregnant women**

The WHO updated their guidelines to allow pregnant women to recieve oral antivirals for the first time in January 2023 [59]. The new guidelines acknowledge there are unquantified risks with providing pregnant women with oral antivirals. Regardless, the updated WHO guidelines stated that oral antivirals should be avaliable to pregnant women through consulation with their healthcare provider since pregnant women with COVID-19 are at 2.4 times increased risk of severe maternal outcomes, and at increased risk of adverse pregancy outcomes including aOR stillbirth (1.8 aOR) and preterm delivery (1.5 aOR) [60].

Here, we present additional results for the prioritisation of pregnant women compared to adults of similar age (Figure S2.4). Providing oral antivirals to pregnant women has a larger impact than providing oral antivirals to non-pregnant adults their age, but a smaller impact than providing these antivirals to older adults and adults with comorbidities (Figure 2 main paper). This is due to pregnant women being a young cohort.


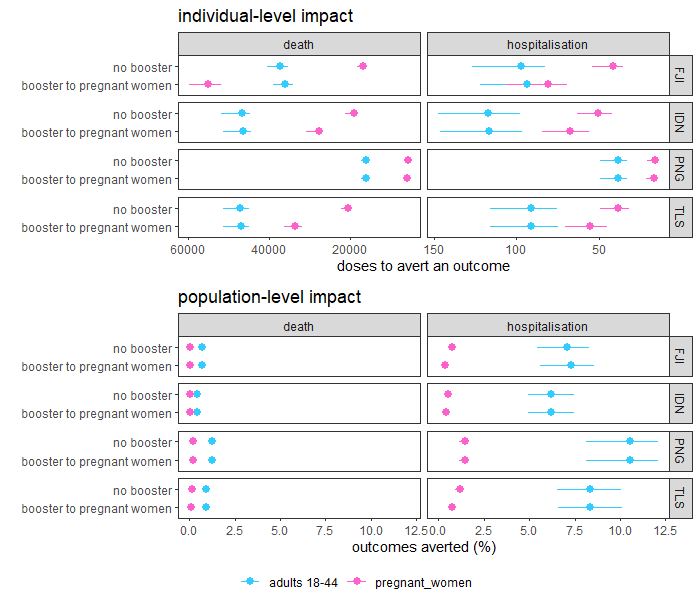


**Figure S2.4 Comparison between the impact of providing oral antivirals to pregnant women and providing oral antivirals to adults 18 to 44 who are not pregnant.**

### **S2.2.3 Use of an oral antiviral with lower effectiveness (molnupiravir)**

Figure S2.5 compares the individual- and population-level impact of molnupiravir and nirmatrelvir-ritonavir. Note that this figure includes severe disease instead of hospitalisation, since there is no observed effect of molnupiravir on the risk of hospitalisation [61].

**
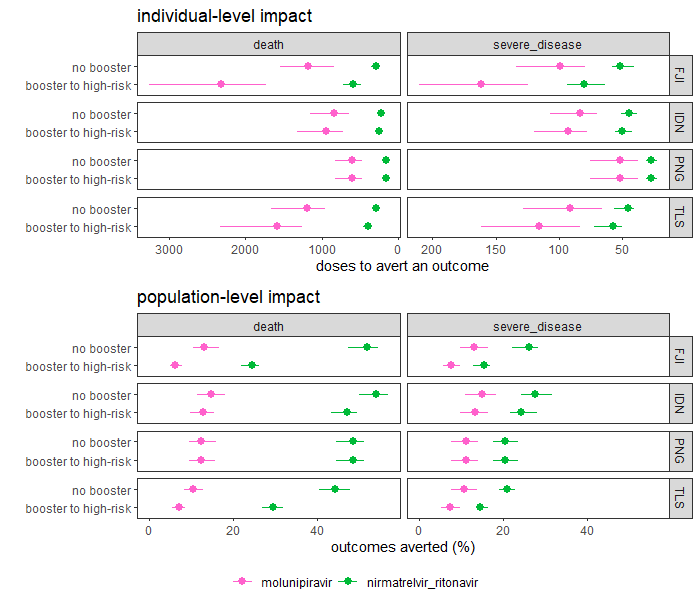
Figure S2.5 Comparison of the impact of providing nirmatrelvir-ritonavir (‘Paxlovid’) and molnupiravir (‘Lagevrio’) to high-risk adults.**

# Reference List

[1] Bilgin GM, Lokuge K, Jabbie E, Munira SL, Glass K. COVID-19 vaccination strategies in settings with limited rollout capacity: a mathematical modelling case study in Sierra Leone. [preprint] avaliable at Research Square. 2023. https://doi.org/10.21203/rs.3.rs-2460525/v1.

[2] Li B, Deng A, Li K, Hu Y, Li Z, Shi Y, et al. Viral infection and transmission in a large, well-traced outbreak caused by the SARS-CoV-2 Delta variant. Nature Communications. 2022;13:460. https://doi.org/10.1038/s41467-022-28089-y.

[3] Kim D, Ali ST, Kim S, Jo J, Lim J-S, Lee S, et al. Estimation of Serial Interval and Reproduction Number to Quantify the Transmissibility of SARS-CoV-2 Omicron Variant in South Korea. Viruses. 2022;14:533.

[4] Hay JA, Kissler SM, Fauver JR, Mack C, Tai CG, Samant RM, et al. Viral dynamics and duration of PCR positivity of the SARS-CoV-2 Omicron variant. medRxiv [preprint]. 2022. https://doi.org/10.1101/2022.01.13.22269257.

[5] United Nations Department of Economic and Social Affairs Population Division. World Population Prospects 2019. Online Edition.

[6] Prem K, Zandvoort KV, Klepac P, Eggo RM, Davies NG, Centre for the Mathematical Modelling of Infectious Diseases C-WG, et al. Projecting contact matrices in 177 geographical regions: An update and comparison with empirical data for the COVID-19 era. PLoS Comput Biol. 2021;17:e1009098. https://doi.org/10.1371/journal.pcbi.1009098.

[7] Hale T, Angrist N, Goldszmidt R, Kira B, Petherick A, Phillips T, et al. A global panel database of pandemic policies (Oxford COVID-19 Government Response Tracker). Nat Hum Behav. 2021;5:529-38. https://doi.org/10.1038/s41562-021-01079-8.

[8] Clark A, Jit M, Warren-Gash C, Guthrie B, Wang HHX, Mercer SW, et al. Global, regional, and national estimates of the population at increased risk of severe COVID-19 due to underlying health conditions in 2020: a modelling study. Lancet Glob Health. 2020;8:e1003-e17. https://doi.org/10.1016/S2214-109X(20)30264-3.

[9] Ayoub HH, Mumtaz GR, Seedat S, Makhoul M, Chemaitelly H, Abu-Raddad LJ. Estimates of global SARS-CoV-2 infection exposure, infection morbidity, and infection mortality rates in 2020. Glob Epidemiol. 2021;3:100068. https://doi.org/10.1016/j.gloepi.2021.100068.

[10] Ferguson N, Ghani A, Hinsley W, Volz E. Report 50 - Hospitalisation risk for Omicron cases in England. In: London IC, editor. Imperial College London: Imperial College London; 2021.

[11] Lin L, Liu Y, Tang X, He D. The Disease Severity and Clinical Outcomes of the SARS-CoV-2 Variants of Concern. Front Public Health. 2021;9:775224. https://doi.org/10.3389/fpubh.2021.775224.

[12] Davies NG, Klepac P, Liu Y, Prem K, Jit M, group CC-w, et al. Age-dependent effects in the transmission and control of COVID-19 epidemics. Nat Med. 2020;26:1205-11. https://doi.org/10.1038/s41591-020-0962-9.

[13] Choi Y, Kim JS, Kim JE, Choi H, Lee CH. Vaccination Prioritization Strategies for COVID-19 in Korea: A Mathematical Modeling Approach. Int J Environ Res Public Health. 2021;18. https://doi.org/10.3390/ijerph18084240.

[14] Gozzi N, Bajardi P, Perra N. The importance of non-pharmaceutical interventions during the COVID-19 vaccine rollout. PLoS Comput Biol. 2021;17:e1009346. https://doi.org/10.1371/journal.pcbi.1009346.

[15] Saldaña F, Velasco-Hernández JX. Modeling the COVID-19 pandemic: a primer and overview of mathematical epidemiology. SeMA Journal. 2021;79:225-51. https://doi.org/10.1007/s40324-021-00260-3.

[16] Mathieu E, Ritchie H, Ortiz-Ospina E, Roser M, Hasell J, Appel C, et al. A global database of COVID-19 vaccinations. Nature Human Behaviour. 2021;5:947-53. https://doi.org/10.1038/s41562-021-01122-8.

[17] Kementerian Kesehatan Republik Indonesia. Vaksinasi COVID-19 Nasional (2022). https://vaksin.kemkes.go.id/#/vaccines. Accessed 25/01/2023.

[18] Jakarta Smart City. Dashboard Cakupan Vaksinasi Fasilitas Kesehatan di Kecamatan (2023). https://corona.jakarta.go.id/en/cakupan-vaksinasi. Accessed 23/01/2023.

[19] Ministry of Health and Medical Services. Nationwide COVID-19 Vaccination Campaign: 28th November 2022 (2022). https://www.health.gov.fj/covid-19-vaccination-campaign/. Accessed 15/12/2022.

[20] Ministry of Health and Medical Services. Public Advisory: COVID-19 Vaccine Booster Doses (2022). https://www.health.gov.fj/booster-doses/. Accessed 15/12/2022.

[21] Ministry of Health and Medical Services. COVID-19 Vaccination for Children Aged 15-17 years old: 7th January 2022 (2022). https://www.health.gov.fj/vaccination-schedule-12-17/. Accessed 15/12/2022.

[22] Ministry of Health and Medical Services. A second booster dose is available to those eligible (2022). https://www.health.gov.fj/second-booster-dose/. Accessed 15/12/2022.

[23] Ministry of Health and Medical Services. Coronavirus (COVID-19) Vaccines (2022). https://www.health.gov.fj/covid-vaccine/vaccine-faqs/. Accessed 15/12/2022.

[24] National Department of Health. Monday, 31st October 2022 COVID-19 vaccination update Papua New Guinea (2022). https://covid19.info.gov.pg/Covax%20Updates/COVAX%20update%20%23263_31102022.pdf. Accessed 23/12/2022.

[25] National Department of Health. PNG NDOH/NCC COVID-19 Vaccination Dashboard (2022). https://covid19.info.gov.pg/files/Situation%20Report/NDoH-Vaccination-Dashboard/_COVID-19_VAC_Dashboard-1%20%281%29%2028%20november.pdf. Accessed 23/12/2022.

[26] World Health Organisation Timor-Leste. Novel Coronavirus (2019-nCoV) situation reports (2022). https://www.who.int/timorleste/emergencies/novel-coronavirus-2019/novel-coronavirus-(2019-ncov)-situation-reports. Accessed 18/01/2023.

[27] United Nation's Children's Fund. COVID-19 Market Dashboard (2022). https://www.unicef.org/supply/covid-19-market-dashboard. Accessed 15/12/2022.

[28] International Vaccine Access Center, World Health Organisation. Results of COVID-19 Vaccine Effectiveness Studies: An Ongoing Systematic Review. 2022.

[29] Andrews N, Stowe J, Kirsebom F, Toffa S, Rickeard T, Gallagher E, et al. Covid-19 Vaccine Effectiveness against the Omicron (B.1.1.529) Variant. N Engl J Med. 2022;386:1532-46. https://doi.org/10.1056/NEJMoa2119451.

[30] Cerqueira-Silva T, de Araujo Oliveira V, Paixao ES, Junior JB, Penna GO, Werneck GL, et al. Duration of protection of CoronaVac plus heterologous BNT162b2 booster in the Omicron period in Brazil. Nat Commun. 2022;13:4154. 10.1038/s41467-022-31839-7.

[31] Cerqueira-Silva T, Katikireddi SV, de Araujo Oliveira V, Flores-Ortiz R, Junior JB, Paixao ES, et al. Vaccine effectiveness of heterologous CoronaVac plus BNT162b2 in Brazil. Nat Med. 2022;28:838-43. https://doi.org/10.1038/s41591-022-01701-w.

[32] Voko Z, Kiss Z, Surjan G, Surjan O, Barcza Z, Wittmann I, et al. Effectiveness and Waning of Protection With Different SARS-CoV-2 Primary and Booster Vaccines During the Delta Pandemic Wave in 2021 in Hungary (HUN-VE 3 Study). Front Immunol. 2022;13:919408. https://doi.org/10.3389/fimmu.2022.919408.

[33] Jara A, Undurraga EA, Zubizarreta JR, González C, Pizarro A, Acevedo J, et al. Effectiveness of homologous and heterologous booster doses for an inactivated SARS-CoV-2 vaccine: a large-scale prospective cohort study. The Lancet Global Health. 2022;10:e798-e806. https://doi.org/10.1016/S2214-109X(22)00112-7.

[34] Tseng HF, Ackerson BK, Luo Y, Sy LS, Talarico CA, Tian Y, et al. Effectiveness of mRNA-1273 against SARS-CoV-2 Omicron and Delta variants. Nature Medicine. 2022;28:1063-71. https://doi.org/10.1038/s41591-022-01753-y.

[35] Sritipsukho P, Khawcharoenporn T, Siribumrungwong B, Damronglerd P, Suwantarat N, Satdhabudha A, et al. Comparing real-life effectiveness of various COVID-19 vaccine regimens during the delta variant-dominant pandemic: a test-negative case-control study. Emerging Microbes & Infections. 2022;11:585-92. https://doi.org/10.1080/22221751.2022.2037398.

[36] Suarez Castillo M, Khaoua H, Courtejoie N. Vaccine-induced and naturally-acquired protection against Omicron and Delta symptomatic infection and severe COVID-19 outcomes, France, December 2021 to January 2022. Eurosurveillance. 2022;27:2200250. https://doi.org/10.2807/1560-7917.ES.2022.27.16.2200250.

[37] Buchan SA, Chung H, Brown KA, Austin PC, Fell DB, Gubbay JB, et al. Estimated Effectiveness of COVID-19 Vaccines Against Omicron or Delta Symptomatic Infection and Severe Outcomes. JAMA Network Open. 2022;5:e2232760-e. https://doi.org/10.1001/jamanetworkopen.2022.32760.

[38] Cerqueira-Silva T, Shah SA, Robertson C, Sanchez MN, Katikireddi SV, de Araújo Oliveira V, et al. Waning of mRNA Boosters after Homologous Primary Series with BNT162b2 or ChadOx1 Against Symptomatic Infection and Severe COVID-19 in Brazil and Scotland: A Test-Negative Design Case-Control Study. SSRN Electronic Journal [preprint]. 2022. https://doi.org/10.2139/ssrn.4082927.

[39] Stowe J, Andrews N, Kirsebom F, Ramsay M, Bernal JL. Effectiveness of COVID-19 vaccines against Omicron and Delta hospitalisation, a test negative case-control study. Nat Commun. 2022;13:5736. 10.1038/s41467-022-33378-7.

[40] Smid M, Berec L, Pribylova L, Majek O, Pavlik T, Jarkovsky J, et al. Protection by Vaccines and Previous Infection Against the Omicron Variant of Severe Acute Respiratory Syndrome Coronavirus 2. J Infect Dis. 2022;226:1385-90. https://doi.org/10.1093/infdis/jiac161.

[41] Ranzani OT, Hitchings MDT, de Melo RL, de Franca GVA, Fernandes CFR, Lind ML, et al. Effectiveness of an inactivated Covid-19 vaccine with homologous and heterologous boosters against Omicron in Brazil. Nat Commun. 2022;13:5536. https://doi.org/10.1038/s41467-022-33169-0.

[42] Gram MA, Emborg H-D, Schelde AB, Friis NU, Nielsen KF, Moustsen-Helms IR, et al. Vaccine effectiveness against SARS-CoV-2 infection or COVID-19 hospitalization with the Alpha, Delta, or Omicron SARS-CoV-2 variant: A nationwide Danish cohort study. PLOS Medicine. 2022;19:e1003992. https://doi.org/10.1371/journal.pmed.1003992.

[43] Hansen CH, Schelde AB, Moustsen-Helm IR, Emborg H-D, Krause TG, Mølbak K, et al. Vaccine effectiveness against SARS-CoV-2 infection with the Omicron or Delta variants following a two-dose or booster BNT162b2 or mRNA-1273 vaccination series: A Danish cohort study. medRxiv [preprint]. 2021:2021.12.20.21267966. https://doi.org/10.1101/2021.12.20.21267966.

[44] Jara A, Cuadrado C, Undurraga EA, García C, Nájera M, Bertoglia MP, et al. Effectiveness and duration of a second COVID-19 vaccine booster. medRxiv [preprint]. 2022:2022.10.03.22280660. https://doi.org/10.1101/2022.10.03.22280660.

[45] Tseng HF, Ackerson BK, Bruxvoort KJ, Sy LS, Tubert JE, Lee GS, et al. Effectiveness of mRNA-1273 vaccination against SARS-CoV-2 omicron subvariants BA.1, BA.2, BA.2.12.1, BA.4, and BA.5. Nature Communications. 2023;14:189. https://doi.org/10.1038/s41467-023-35815-7.

[46] Grewal R, Nguyen L, Buchan SA, Wilson SE, Costa AP, Kwong JC. Effectiveness and Duration of Protection of a Fourth Dose of COVID-19 mRNA Vaccine among Long-Term Care Residents in Ontario, Canada. The Journal of Infectious Diseases. 2022. https://doi.org/10.1093/infdis/jiac468.

[47] Canetti M, Barda N, Gilboa M, Indenbaum V, Asraf K, Gonen T, et al. Six-Month Follow-up after a Fourth BNT162b2 Vaccine Dose. New England Journal of Medicine. 2022;387:2092-4. https://doi.org/10.1056/NEJMc2211283.

[48] Fabiani M, Mateo-Urdiales A, Sacco C, Rota MC, Petrone D, Bressi M, et al. Relative effectiveness of a 2nd booster dose of COVID-19 mRNA vaccine up to four months post administration in individuals aged 80 years or more in Italy: A retrospective matched cohort study. Vaccine. 2022. https://doi.org/10.1016/j.vaccine.2022.11.013.

[49] Khare S, Gurry C, Freitas L, Schultz MB, Bach G, Diallo A, et al. GISAID's Role in Pandemic Response. China CDC Wkly. 2021;3:1049-51. https://doi.org/10.46234/ccdcw2021.255.

[50] Mullen KM, Ardia D, Gil DL, Windover D, Cline J. DEoptim: An R Package for Global Optimization by Differential Evolution. Journal of Statistical Software. 2011;40:1 - 26. https://doi.org/10.18637/jss.v040.i06.

[51] Ariawan I, Jusril H, Farid MN, Riono P, Wahyuningsih W, Handayani DOTL, et al. SARS-CoV-2 Antibody Seroprevalence in Jakarta, Indonesia. Kesmas. 2022;17:169-74. https://doi.org/10.21109/kesmas.v17i3.6070.

[52] Ahmad RA, Indriani C, Arisanti RR, Nanda RO, Mahendradhata Y, Wibawa T. Seroprevalence of SARS-CoV-2 and risk factors in Bantul Regency, Yogyakarta, Indonesia. medRxiv [preprint]. 2022:2022.06.07.22276128. https://doi.org/10.1101/2022.06.07.22276128.

[53] Herlinda O, Bella A, Kusnadi G, Swasthika Nurshadrina D, Thoriq Akbar M, Nida S, et al. Seroprevalence of antibodies against SARS-Cov-2 in the high impacted sub-district in Jakarta, Indonesia. PLOS ONE. 2021;16:e0261931. https://doi.org/10.1371/journal.pone.0261931.

[54] Lusida MI, Gunawan E, Megasari NLA, Yamani LN, Juniastuti, Utsumi T, et al. Viral shedding and the durability of immunoglobulin G antibodies to severe acute respiratory syndrome coronavirus 2. Microbiology and Immunology. 2022;66:173-8. https://doi.org/10.1111/1348-0421.12962.

[55] Scarfo M. Extreme weather and COVID-19 collide in Timor-Leste. https://www.unicef.org.au/stories/flooding-and-covid-19-timor-leste2021.

[56] Jamie Tahana. 'We don't have any grasp': Covid-19 crisis pushes PNG hospitals to the brink (2021). https://www.rnz.co.nz/international/pacific-news/441445/we-don-t-have-any-grasp-covid-19-crisis-pushes-png-hospitals-to-the-brink. Accessed 20/01/2023.

[57] Bergeri I, Whelan MG, Ware H, Subissi L, Nardone A, Lewis HC, et al. Global SARS-CoV-2 seroprevalence from January 2020 to April 2022: A systematic review and meta-analysis of standardized population-based studies. PLOS Medicine. 2022;19:e1004107. https://doi.org/10.1371/journal.pmed.1004107.

[58] Dong E, Du H, Gardner L. An interactive web-based dashboard to track COVID-19 in real time. Lancet Infect Dis. 2020;20:533-4. https://doi.org/10.1016/S1473-3099(20)30120-1.

[59] World Health Organization. Therapeutics and COVID-19: living guideline, 13 January 2023. Geneva: World Health Organization; 2023.

[60] Royal College of Obstetricians and Gynaecologists. Coronavirus (COVID-19), infection in pregnancy (2022). https://www.rcog.org.uk/media/xsubnsma/2022-03-07-coronavirus-covid-19-infection-in-pregnancy-v15.pdf. Accessed 19/08/2022.

[61] Wong CKH, Au ICH, Lau KTK, Lau EHY, Cowling BJ, Leung GM. Real-world effectiveness of molnupiravir and nirmatrelvir plus ritonavir against mortality, hospitalisation, and in-hospital outcomes among community-dwelling, ambulatory patients with confirmed SARS-CoV-2 infection during the omicron wave in Hong Kong: an observational study. The Lancet. 2022;400:1213-22. https://doi.org/10.1016/s0140-6736(22)01586-0.
